# Transcriptome analysis of heat stress and drought stress in pearl millet based on Pacbio full-length transcriptome sequencing

**DOI:** 10.1186/s12870-020-02530-0

**Published:** 2020-07-08

**Authors:** Min Sun, Dejun Huang, Ailing Zhang, Imran Khan, Haidong Yan, Xiaoshan Wang, Xinquan Zhang, Jian Zhang, Linkai Huang

**Affiliations:** 1 Department of Grassland Science, Sichuan Agricultural University, Chengdu, 6111130 China; 2 Herbivorous Livestock Research Institute, Chongqing Academy of Animal Sciences, Chongqing, China; 3 Department of Horticulture, Virginia Tech, Blacksburg, VA 24061 USA

**Keywords:** Pearl millet, Pacbio sequencing, Illumina sequencing, Heat stress, Drought stress

## Abstract

**Background:**

Heat and drought are serious threats for crop growth and development. As the sixth largest cereal crop in the world, pearl millet can not only be used for food and forage but also as a source of bioenergy. Pearl millet is highly tolerant to heat and drought. Given this, it is considered an ideal crop to study plant stress tolerance and can be used to identify heat-resistant genes.

**Results:**

In this study, we used Pacbio sequencing data as a reference sequence to analyze the Illumina data of pearl millet that had been subjected to heat and drought stress for 48 h. By summarizing previous studies, we found 26,299 new genes and 63,090 new transcripts, and the number of gene annotations increased by 20.18%. We identified 2792 transcription factors and 1223 transcriptional regulators. There were 318 TFs and 149 TRs differentially expressed under heat stress, and 315 TFs and 128 TRs were differentially expressed under drought stress. We used RNA sequencing to identify 6920 genes and 6484 genes differentially expressed under heat stress and drought stress, respectively.

**Conclusions:**

Through Pacbio sequencing, we have identified more new genes and new transcripts. On the other hand, comparing the differentially expressed genes under heat tolerance with the DEGs under drought stress, we found that even in the same pathway, pearl millet responds with a different protein.

## Background

Currently, dramatic climate change, including severe heat and drought, are threatening agricultural production [1]. According to the report by the Intergovernmental Panel on Climate Change (IPCC), the global temperature is expected to rise 0.2 °C per decade, and by 2100, it will be 1.8 to 4.0 °C higher than the current level. One previous study revealed that every 1 °C increase in the global average temperature is reducing the yield of major agricultural crops by the following amounts: wheat 6%, rice 3.2%, corn 7.4%, and soybean 3.1% [2]. The average precipitation in many mid-latitude and subtropical arid regions is expected to decrease and severely affect the crop yield. A report by the United Nations Environment Program showed that the crop yields in semi-arid and arid regions were reduced by approximately 3.6 billion hectares (25% of the world’s highlands).

Pearl millet (*Pennisetum glaucum* (L.) R. Br.) ranks 6th behind the world’s most important cereal crops, such as wheat, rice, maize, sorghum and barley [3], and it is grown on an area of 31 million hectare (ICRISAT 2016) in the world. Pearl millet is an ideal plant for bioethanol production and can be used as a sustainable and alternative energy source, due to its high concentration of easily extractable fermentable sugar.

Pearl millet is highly tolerant to heat because its root branchings and flowering speed increase along with the growth of new leaves at certain high temperature levels [4]. The relative growth rate and net assimilation rate (NAR) significantly increase in pearl millet and slightly decrease in maize subjected to high temperature (38/27 °C) [5]. Previous reports distinguish pearl millet as a crop that is highly resistant toward drought. Under a certain degree of drought stress, pearl millet has a higher resistance to drought than other species of millet (finger millet, Job’s tears, barnyard millet, common millet, and foxtail millet) and maintains an unchanged morphology, particularly with respect to the leaf area and shoot fresh and dry weight [6, 7]. Therefore, pearl millet is an ideal material to study the mechanism of heat and drought resistance of cereal crops. Studies on the root activity [4], seed germination rate [8] and seedling growth rate of pearl millet under heat stress [9] have previously been reported. Some researchers have cloned and investigated the HSP (sHSP [10], hsp70 [11] and HSP90 [12]) genes in pearl millet, but the presence of only a few reports do not provide sufficient information on the sequencing of pearl millet under heat stress. In addition, studies on the sequencing of pearl millet under drought stress are still limited [13, 14].

The genome of pearl millet was reported in 2017 [15], but short reads cannot be mapped to the genes due to incomplete genome annotations. Single-molecule real-time sequencing technology also known as the next-generation sequencing, such as PacBio sequencing, allows the direct production of full-length transcripts, making it ideal for transcript recovery and isoform detection of well-sequenced and/or incomplete genomic sequences [16, 17], but its disadvantage is low throughput [18]. Second-generation sequencing using the Illumina platform is an effective way to quantify the gene expression and high-quality reads. However, due to the short length of the reads produced by the second generation of sequencing, computational assembly is required [18, 19]. To avoid these problems, we combined the two sequencing techniques to study the similarities and differences of the molecular mechanism of the response of pearl millet under heat stress and drought stress. First, we used Illumina sequencing data to correct the raw data of the full-length transcriptome, and second, we used the corrected full-length transcriptome data as a reference sequence to analyze the short sequencing data.

In this study, we obtained the following results: a. We identified 63,090 new transcripts and 26,299 new genes; b. Compared with previous studies, the annotation rate of pearl millet genes increased by 20.18%; c. The heat shock protein under HSPs still function after 48 h of heat stress; d. The plants can still be regulated by the abscisic acid (ABA) pathway under 48 h drought stress; e. Even with the same mechanism of response, plants tend to select different protein species when they face different stresses. This study demonstrated the changes of pearl millet under 48 h heat stress and drought stress at the molecular level, which provided a new concept to study the tolerance of pearl millet. In addition, the new genes that were discovered provide more resources for breeding projects.

## Results

### A total of 132,488 non-redundant transcripts and 64,878 genes were identified by full-field transcriptome modeling

Acquisition of the full-length transcriptome data was based on the third-generation sequencing platform of PacBio Sequel. By filtering the raw data, we removed the connector and original offline data that was less than 50 bp in length to obtain 6,842,837 Subreads based on 17.89 G (Table 1). The average length of the Subreads was 2615 bp. A total of 354,139 circular consensus (CCS) were obtained through conditional screening (full passes of 1 and quality of 0.80). Finally, 306,369 full length non-chimeric reads (Flnc) were obtained with complete 5′-primers, 3′-primers and poly-A tails in which an average of the Flnc read length was 2897.

**Table 1:** Summary of PacBio transcripts and comparisons with genomes reported in 2017

	Pacbio		Varshney et al., 2017
	Before correct	After correct	
Subreads base(G)	17.89		
Subreads number	6,842,837		
Average subreads length	2615		
N50(subreads)	2787		
CCS	354,139		
5′-primer	332,357		
3′-primer	332,288		
Poly-A	326,534		
Full length	306,369		
Flnc	303,627		
Average flnc read length	2897		
Consensus reads	132,488		
Total_nucleotides	409,482,590	410,858,545	
Total_number	132,488	132,488	
Mean_length	3091	3102	1014.71
Min_length	200	201	
Max_length	14,226	14,277	
N50(consensus)	3288	3302	
N90(consensus)	1999	2006	
Number of transcripts		132,488	69,398
Number of Genes		64,878	38,579
Number of genes annotated		62,436(96.24%)	29,344 (76.06%)
Number of genes unannotated		2442(3.76%)	9235 (23.94%)

The third-generation sequencing technology, represented by PacBio, has the advantage of long read lengths, but the technique has a high single base error rate. To reduce the high error rate, the Illumina data were used for correction. After correcting the data, 132,588 consensus, 3302 N50, and 2006 N90 were obtained. CD-HIT software removed redundant and similar sequences that resulted in 132,488 non-redundant transcripts and 64,878 genes.

### Annotation of 62,436 (96.24%) genes

**Fig. 1:**
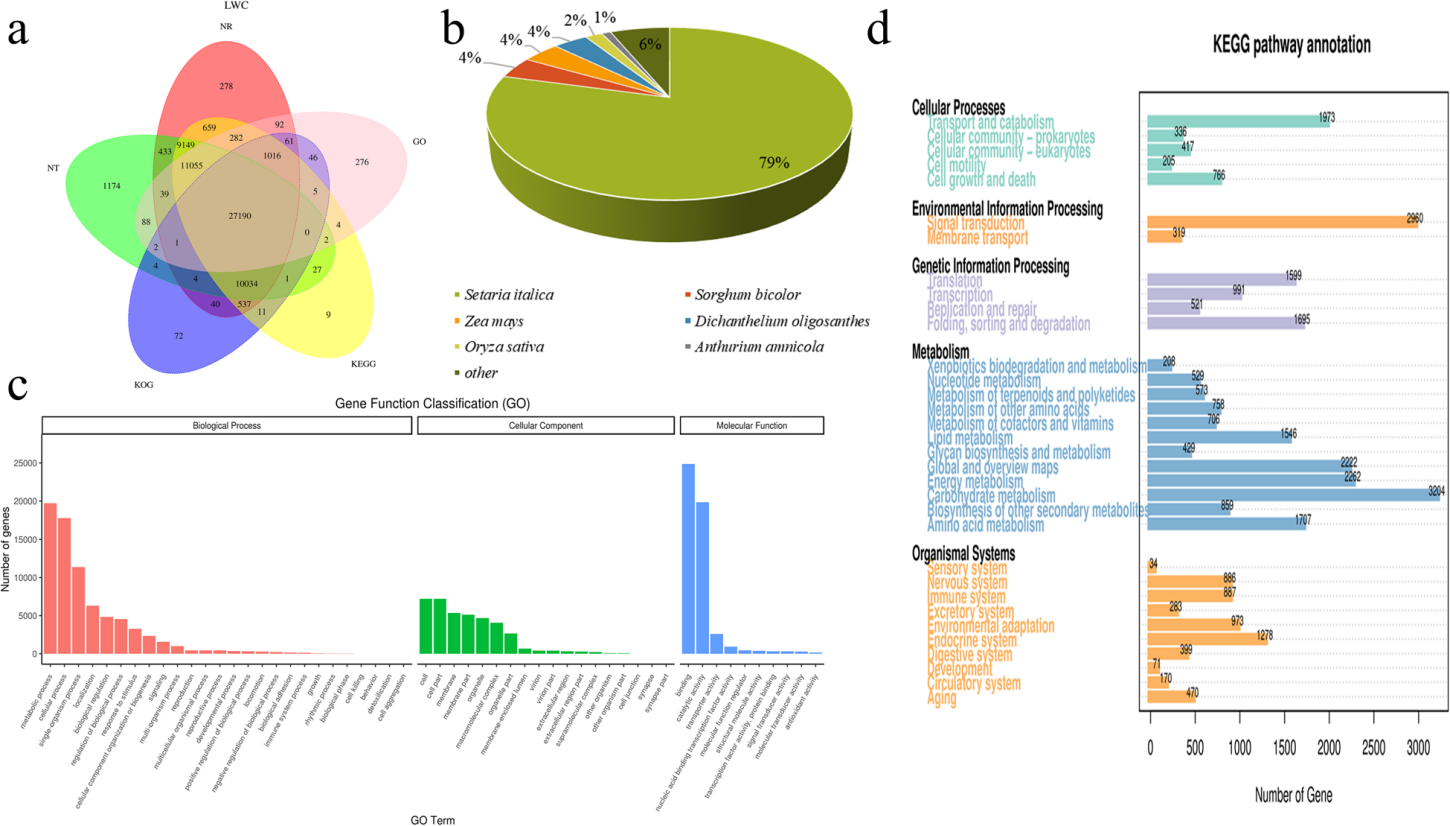
Annotation of pearl millet transcript. **a** Gene function annotations in 5 databases (Nr, Nt, GO, KEGG, KOG). **b** Homologous **species** distribution of pearl millet annotated in the NR database. **c** Annotation of the GO function of the pearl millet transcript. **d** Annotation of the KEGG function of the pearl millet transcript

To obtain a comprehensive genetic annotation, we analyzed the total of the 64,878 genes using NCBI nucleotide sequences (Nt) by BLASTN (E-value 1^e-10^), NCBI non-redundant protein sequence (Nr), Protein family (Pfam), eukaryotic Ortholog Groups (KOG), Swiss-prot, Kyoto Encyclopedia of Genes and Genomes (KEGG), Gene Ontology (GO), and BLASTx (E-value 1^e-10^) databases. The Venn diagram demonstrated that 27,190 genes were simultaneously annotated in all the databases (Nr, Nt, Pfam, GO, KEGG, KOG, and Swiss-prot) (Fig. 1a). There were 62,436 genes that were annotated in at least one database of which 60,870 (93.82%) genes were annotated by the NR database. Based on the alignment of sequence homology, the result showed that 48,226 (74.33%) sequences were found against *Setaria italica*; 2471 (3.81%) sequences had significant hits for *Sorghum bicolor*, followed by *Zea mays* (2422, 3.73%), *Dichanthelium oligosanthes* (2218, 3.42%) and *Oryza sativa* (1100, 1.70%). A total of 13.01% of the sequences were homologous to other species (Fig. 1b).

A total of 40,159 (61.90%) genes were annotated as GO terms. The results of the GO enrichment analysis showed that the genes were primarily enriched in metabolic process, cellular process, single−organism process, cell, cell part, membrane, binding, catalytic activity, transporter activity in biological process (BP), molecular function (MF), and cellular component (CC) (Fig. 1c).

A total of 59,981 (92.45%) genes were annotated in the KEGG database (Fig. 1d). The KEGG pathway analysis revealed that 3204 (4.94%) genes were clustered in the carbohydrate metabolism pathway, 2960 (4.56%) genes in the signal transduction pathway, and 2262 (3.49%) in energy metabolism.

We annotated 39,024 (60.15%) genes in the KOG database. A total of 7332 (11.30%) genes were annotated in general function prediction only, while 5256 (8.10%) were in signal transduction mechanisms and 3997 (6.16%) in post-translational modification, protein turnover and chaperones.

### A total of 2792 TFs, 1223 TRs, 694 lncRNA, and 1124 alternative splicing events were identified in pearl millet

**Fig. 2:**
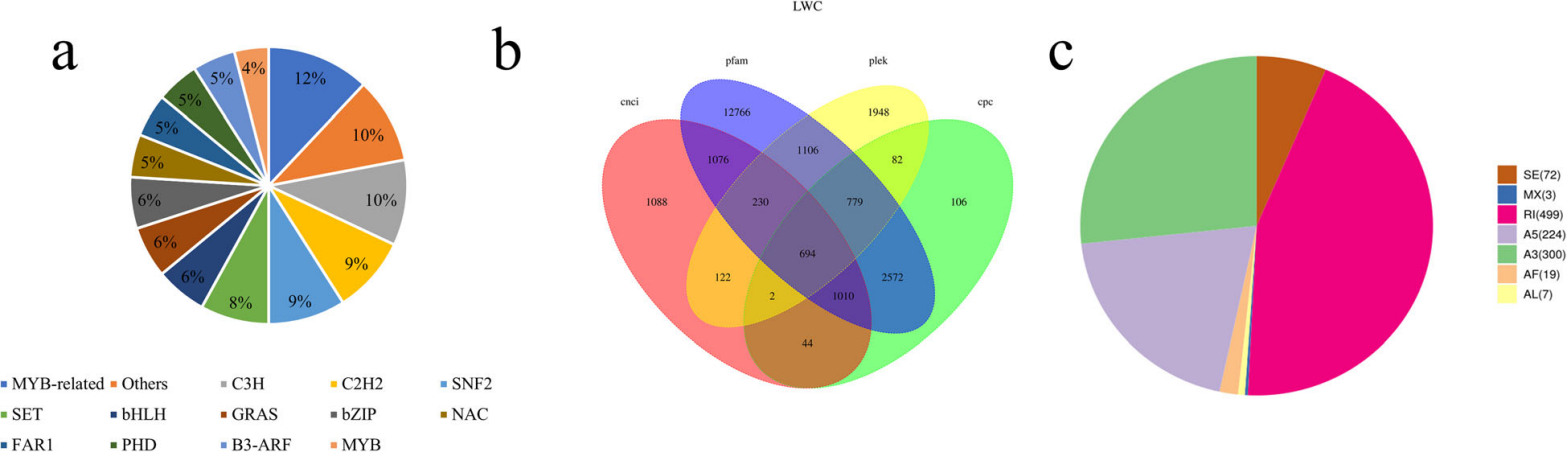
Prediction of transcription factors, long-non-coding RNAs and alternative splicing of pearl millet transcripts. **a** Transcription factor statistics predicted by iTAK. **b** Venn diagram of the number of lncRNAs predicted by Calculator (CPC), Coding-Non-Coding Index (CNCI), Coding Potential Assessment Tool (CPAT), and pfam protein structure domain analysis. **c** Prediction of alternative splicing events by SUPPA

By predicting 132,488 non-redundant transcripts using iTAK software, 2792 genes were predicted to be TFs, and 1223 genes were predicted to be TRs (Fig. 2a). A total of 272 transcripts were predicted to be MYB-related TFs. MYB-associated transcription factors are important telomere-binding proteins that have the effect of maintaining the integrity of the chromosomal structure and regulating gene transcription, and 235 transcripts were predicted as other TFs. A total of 196 transcription factors were considered C3H transcription factors, which are involved in abiotic stress responses in plants [20]. There are 190 transcripts that were anchored as C2H2 TFs, primarily related to plant growth and development and the response to environmental stresses [21].

In addition, CNCI, CPC, Pfam-scan and PLEK tools were used to predict long non-coding RNA (LncRNA) (Fig. 2b). Based on known annotations, 4266 transcripts were detected as long non-coding proteins through CNCI. Consistent with the NCBI database, 5389 transcripts were predicted as long non-coding proteins by CPC (e-value 1^e-10^). Translation and identification of transcripts via Pfam-scan (default parameters of -E 0.001 --domE 0.001) showed that 20,233 transcripts were long non-coding proteins. There were 4963 non-coding transcripts belonging to long protein by screening with PLEK (default parameters of -minlength 200), while 694 transcripts were simultaneously screened by CNCI, CPC, Pfam-scan and PLEK.

A total of 1124 alternative splicing events were identified by SUPPA (Fig. 2c). There were 499 genes belonging to the Retained Intron Type, 300 genes as Alternative 3′ Splice Sites, and 224 genes were related to Alternative 5′ Splice Sites.

### Photosynthetic proteins may be involved in resistance to heat stress

There were a total of 6920 differentially expressed genes (DEGs), of which 3555 and 3365 genes were up- and down-regulated, respectively.

**Fig. 3:**
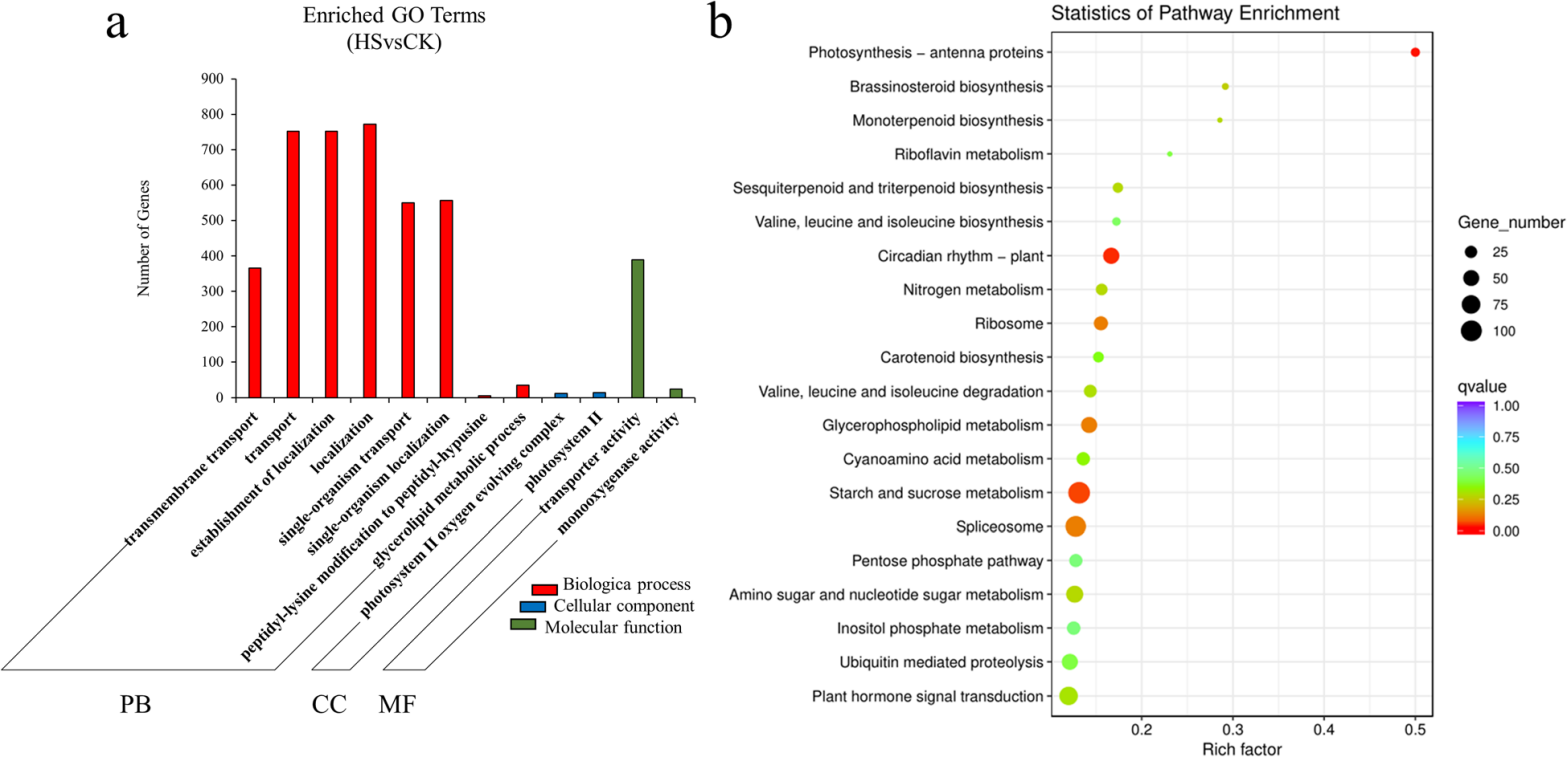
GO analysis and KEGG analysis of DEGs under the heat stress of pearl millet. **a** GO analysis of DEGs under the heat stress of pearl millet. **b** KEGG analysis of DEGs under the heat stress of pearl millet

GO enrichment analysis displayed that “transmembrane transport” (114 up-regulation and 252 down-regulation), “transport” (308 up-regulation and 444 down-regulation) and “establishment of localization” (308 up-regulation and 444 down-regulation) were the most enriched terms of the biological process. “photosystem II oxygen evolving complex” (5 up-regulation and 7 down-regulation) and “photosystem II” (5 up-regulation and 10 down-regulation) were the most enriched terms of the cellular component (Additional file 2). “transporter activity” (141 up-regulation and 248 down-regulation) and “monooxygenase activity” (104 up-regulation and 10 down-regulation) were the most enriched terms of the cellular component. We found that “RNA splicing”, “anatomical structure morphogenesis”, “regulation of translation”, “spliceosomal complex”, “translation regulator activity”, “zinc ion binding” and “nucleic acid binding” were the most enriched GO terms among the differentially expressed up-regulated genes. “single−organism localization”, “single−organism transport”, “transmembrane transport”, “intrinsic component of membrane”, “integral component of membrane”, “membrane part”, “transmembrane transporter activity”, “transporter activity” and “diacylglycerol O−acyltransferase activity” were the most enriched GO terms among the differentially expressed down-regulated genes (Fig. 3a).

The KEGG enrichment analysis of 6920 DEGs showed that “Photosynthesis - antenna proteins”, “Circadian rhythm-plant” and “Starch and sucrose metabolism” were the most enriched pathways. A KEGG enrichment analysis of 3555 up-regulated DEGs revealed that “Spliceosome”, “Valine, leucine and isoleucine degradation” and “Valine, leucine and isoleucine degradation” were the most enriched terms, and a KEGG enrichment analysis of 3365 down-regulated differentially expressed genes indicated that “Photosynthesis - antenna proteins”, “Glycerophospholipid metabolism” and “Circadian rhythm - plant” were the most enriched pathways (Fig. 3b).

### Glycerophospholipid metabolic pathway may play an important role in the drought tolerance of pearl millet

By comparing the gene expression levels under drought treatment with the control conditions, a total of 6484 DEGs (*P* < 0.05) were shown to be up- or down-regulated between samples collected at 48 h. There were 3041 up-regulated and 3443 down-regulated DEGs.

**Fig. 4:**
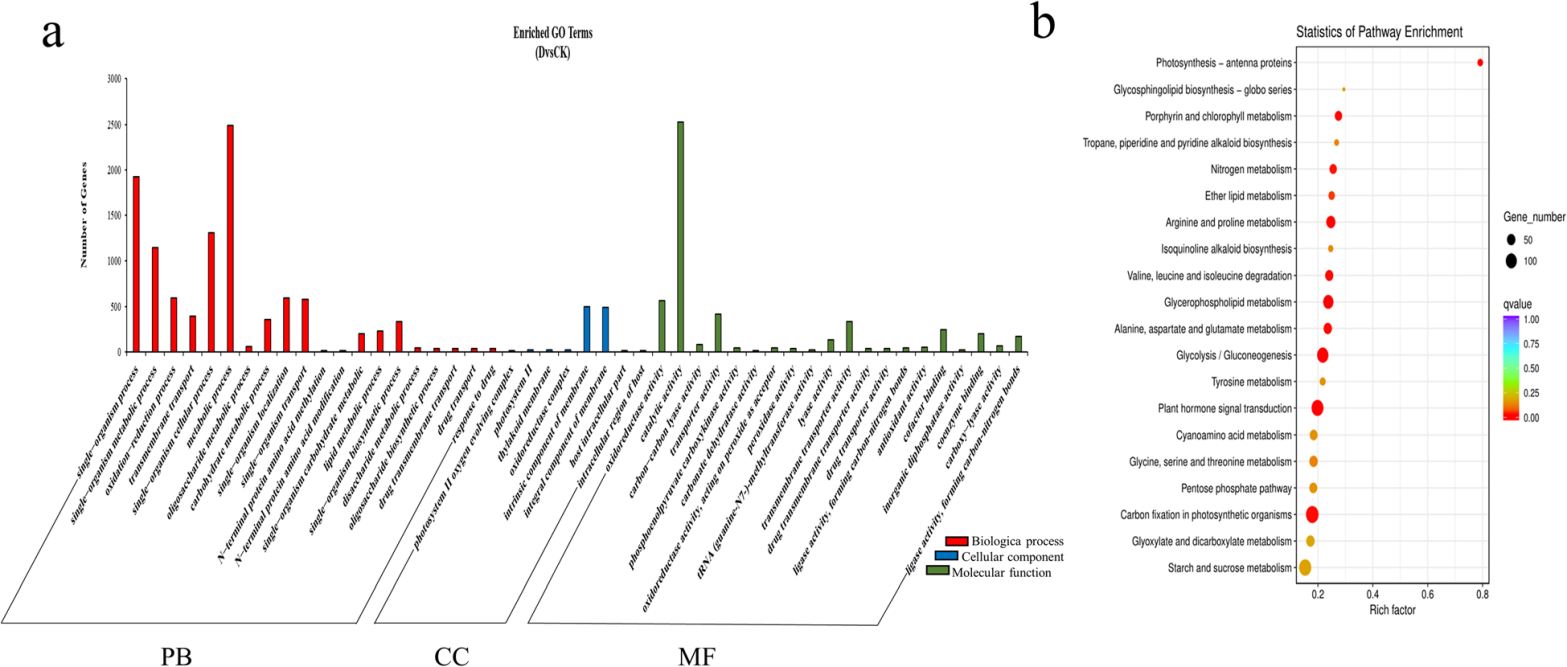
GO analysis and KEGG analysis of DEGs under drought stress of pearl millet. **a** GO analysis of DEGs under drought stress of pearl millet. **b** KEGG analysis of DEGs under drought stress of pearl millet

GO enrichment analysis displayed that “single−organism process” (964 up-regulation and 959 down-regulation), “single−organism metabolic process” (593 up-regulation and 552 down-regulation), “oxidation−reduction process” (289 up-regulation and 303 down-regulation) “photosystem II oxygen evolving complex” (0 up-regulation and 3 down-regulation), “photosystem II” (0 up-regulation and 21 down-regulation), “thylakoid membrane” (0 up-regulation and 18 down-regulation), “oxidoreductase activity” (284 up-regulation and 276 down-regulation), “catalytic activity” (1254 up-regulation and 3165 down-regulation) and “carbon−carbon lyase activity” (81 up-regulation and 17 down-regulation) were the most enriched terms. The results showed that the up-regulated DEGs were primarily enriched in “single−organism process”, “single−organism metabolic process”, “oligosaccharide metabolic process”, “tRNA (guanine−N7−)−methyltransferase activity”, “catalytic activity” and “tRNA (guanine) methyltransferase activity” terms. However, the down-regulated DEGs were primarily enriched in “single−organism process”, “metabolic process”, “protein import into nucleus”, “photosystem II”, “photosystem II oxygen evolving complex”, “thylakoid membrane”, “carbon−carbon lyase activity”, “phosphoenolpyruvate carboxykinase activity” and “lyase activity” (Fig. 4a).

**Fig. 5:**
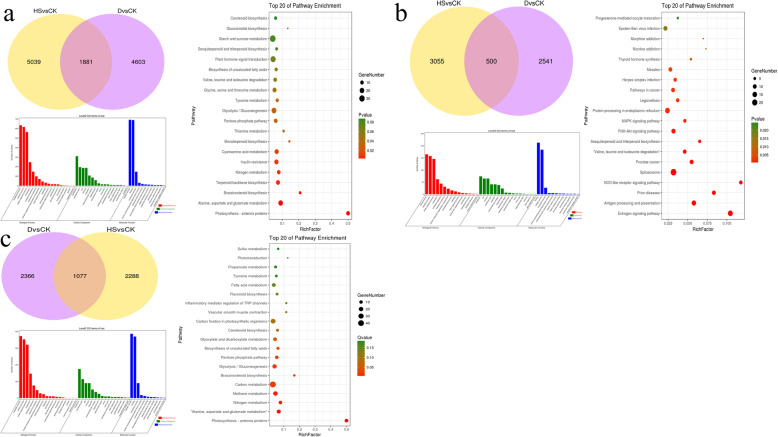
Analysis of DEGs under heat stress and drought stress. **a** Veen of DEGs under heat stress and drought stress and GO analysis and KEGG analysis of DEGs simultaneously present under heat stress and drought stress. **b** Veen up-regulation of DEGs under heat stress and drought stress and GO analysis and KEGG analysis of up-regulated DEGs simultaneously present under heat stress and drought stress. **c** Veen down-regulation of DEGs under heat stress and drought stress and GO analysis and KEGG analysis of down-regulated DEGs simultaneously present under heat stress and drought stress

The results of the KEGG enrichment analysis indicated that the DEGs were primarily enriched in “Photosynthesis - antenna proteins”, “Glycerophospholipid metabolism” (Additional file 3) and “Glycolysis/Gluconeogenesis” pathway. The up-regulated DEGs were primarily enriched in “Glycerophospholipid metabolism”, “Valine, leucine and isoleucine degradation” and “Arginine and proline metabolism” pathway. The down-regulated DEGs were primarily enriched in “Carbon fixation in photosynthetic organisms”, “Photosynthesis - antenna proteins” and “Porphyrin and chlorophyll metabolism” pathways (Fig. 5b).

### A total of 1881 genes were involved in both heat stress and drought stress responses

**Fig. 6:**
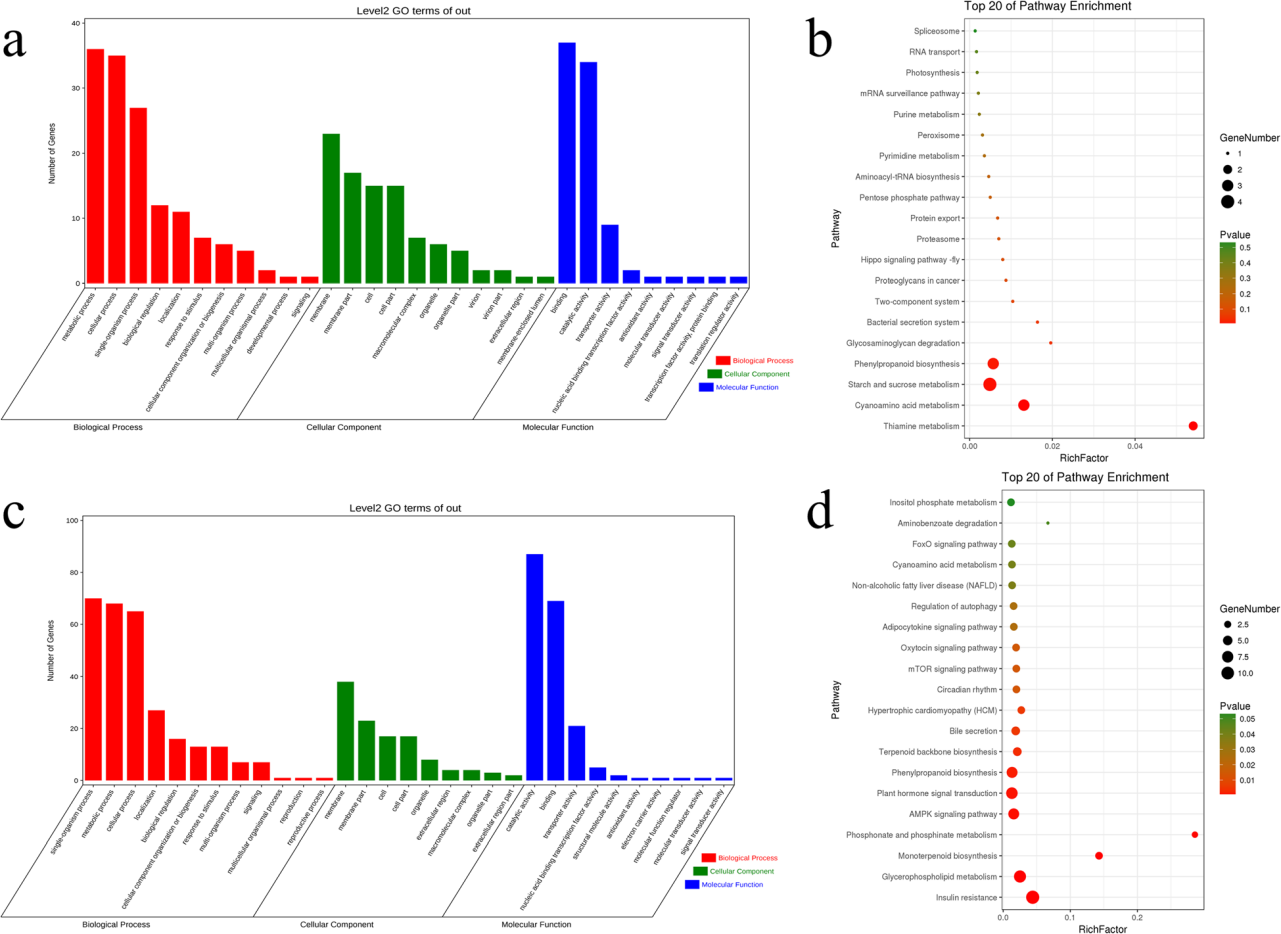
Analysis of DEGs with different expression patterns under heat stress and drought stress. **a** GO analysis of 122 DEGs up-regulated under heat stress but down-regulated under drought stress. **b** GO analysis of 122 DEGs up-regulated under heat stress but down-regulated under drought stress. **c** GO analysis of 182 DEGs down-regulated under heat stress but up-regulated under drought stress. **d** KEGG analysis of 182 DEGs down-regulated under heat stress but up-regulated under drought stress

A total of 1881 DEGs were simultaneously found in both heat stress and drought stress (Fig. 5a). GO enrichment analysis results show that the most enriched GO terms were “metabolic process”, “cellular process”, “membrane”, “membrane part”, “catalytic activity”, and “binding” (Fig. 5a). The KEGG enrichment analysis showed that “Photosynthesis - antenna proteins”, “Alanine, aspartate and glutamate metabolism”, “Brassinosteroid biosynthesis”, and “Terpenoid backbone biosynthesis” were the most enriched pathways (Fig. 5a). Among the 1881 DEGs, 500 genes were up-regulated under both heat stress and drought stress (Fig. 5b). Through GO enrichment analysis, it was found that the 500 DEGs were primarily enriched in “cellular process”, “metabolic process”, “membrane”, “cell”, “binding”, and “catalytic activity” GO terms (Fig. 5b). A KEGG enrichment analysis found that the main enrichments were “Estrogen signaling pathway”, “Antigen processing and presentation”, and “NOD-like receptor signaling pathway” pathway (Fig. 5b). A total of 1077 DEGs were simultaneously down-regulated under heat stress and drought stress (Fig. 5c). GO enrichment analysis showed that the main enrichment was in “metabolic process”, “cellular process”, “membrane”, “membrane part”, “catalytic activity”, and “binding” GO terms (Fig. 5c). A KEGG enrichment analysis found that the main enrichment was in “photosynthesis - antenna protein”, “alanine, aspartic acid and glutamate metabolism”, and “nitrogen metabolism” (Fid 5c). A total of 122 DEGs were up-regulated under heat stress but down-regulated under drought stress. GO enrichment analysis of the 122 DEGs showed that “metabolic process”, “cellular process”, “membrane”, “membrane part”, “binding”, “catalytic activity” were the most enriched GO terms (Fig. 6a). The results of the KEGG enrichment analysis indicated that 122 differentially expressed genes were primarily enriched in the “Thiamine metabolism”, “Cyanoamino acid metabolism”, “Starch and sucrose metabolism”, and “Phenylpropanoid biosynthesis” pathways (Fig. 6b). A total of 182 DEGs were down-regulated under heat stress but were up-regulated under drought stress. GO enrichment analysis of the 182 DEGs revealed that the main enrichment was at the “single-organism process”, “metabolic process”, “membrane”, “membrane part”, “catalytic activity”, and “binding” GO terms (Fig. 6c). The results of a KEGG enrichment analysis indicate that the main enrichment is in the “Glycerophospholipid metabolism”, “Monoterpenoid biosynthesis”, and “Phosphonate and phosphinate metabolism” pathways (Fig. 6d).

**Fig. 7:**
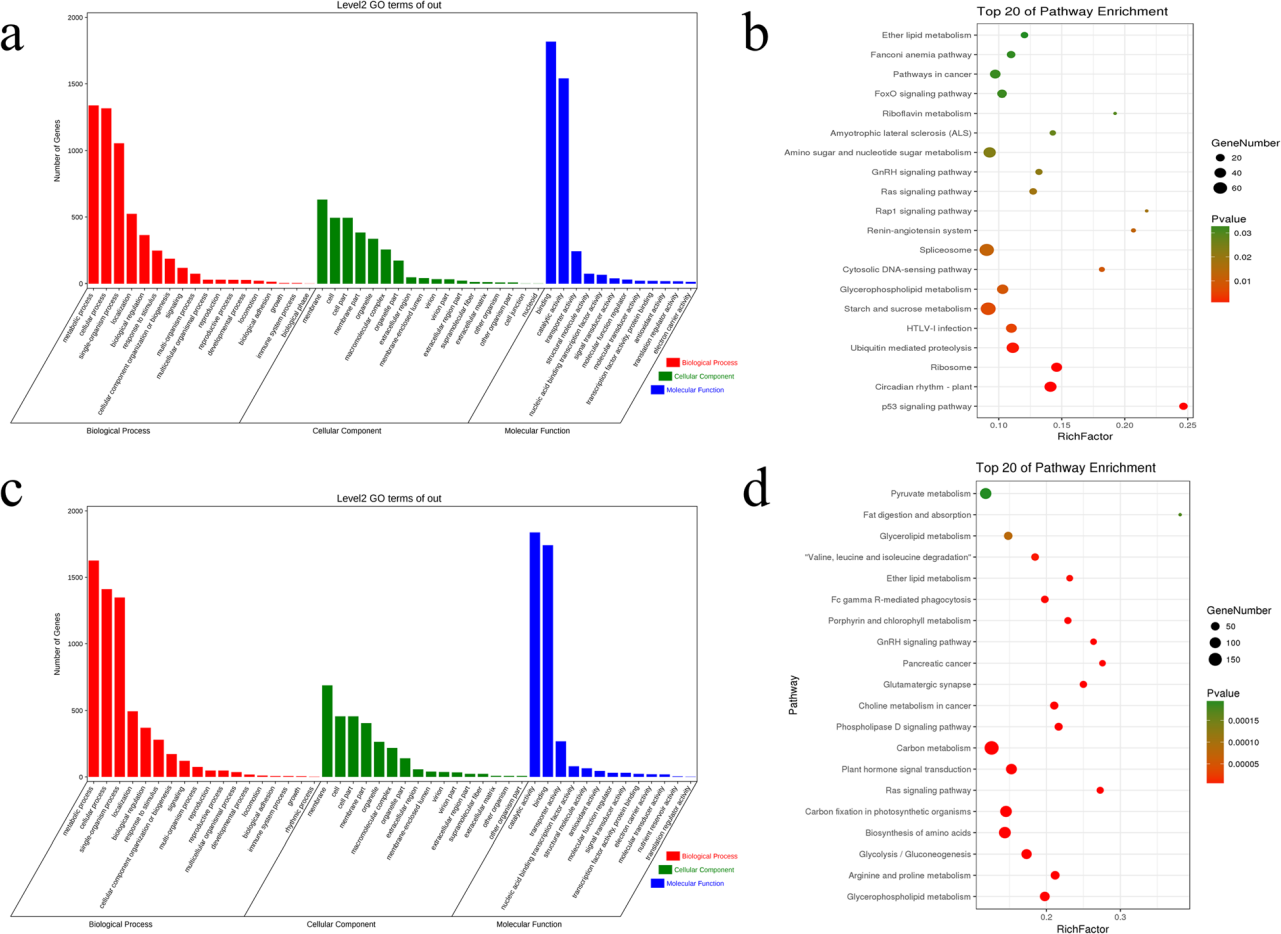
Analysis of DEGs specific to heat stress or drought stress. **a** GO analysis of DEGs specific to heat stress. **b** KEGG analysis of DEGs specific to heat stress. **c** GO analysis of DEGs specific to drought stress. **d** KEGG analysis of DEGs specific to drought stress

There were 5039 and 4603 DEGs specific to heat stress and drought stress, respectively (Fig. 5a). A total of 5039 DEGs analysed by GO enrichment found that these genes were primarily enriched in “metabolic process”, “cellular process”, “membrane”, “cell”, “binding”, and “catalytic activity” (Fig. 7a). A KEGG enrichment analysis showed that 5039 genes were primarily enriched in the “p53 signaling pathway”, “Circadian rhythm - plant”, and “Ribosome” and “Ubiquitin mediated proteolysis” pathways (Fig. 7b). The 4603 DEGs were only found in drought stress enriched in “metabolic process”, “cellular process”, “membrane”, “cell”, “catalytic activity”, and “binding” GO terms (Fig. 7c). A KEGG enrichment analysis showed that 4603 DEGs were primarily enriched in the “Glycerophospholipid metabolism”, “Arginine and proline metabolism”, “Glycolysis/Gluconeogenesis”, and “Biosynthesis of amino acids” pathways (Fig. 7d).

## Discussion

### A total of 63,090 new transcripts, 26,299 new genes were identified, and annotations increased by 20.18%

Pearl millet is a versatile grain that can be used as grain, hay and green fodder. It is distributed in arid and semi-arid regions, such as India and sub-Saharan Africa [22]. In recent years, there have been many reports on the sequencing of corn [23, 24], rice [25] and wheat [26], but only a few that examined pearl millet. Some researchers constructed a genetic linkage map of 640 cM using GBS sequencing (genotyping-by-sequencing) to identify the genes associated with Striga and other agronomic traits [27]. In April 2016, researchers performed an RNA-Seq analysis of pearl millet planted in two climate gradients to explore their adaptability to climate [28]. In May 2016, researchers constructed the highest density genetic linkage map using GBS sequencing. The total average density of SNP markers in the map was 23.23 SNP/cM, and the total length was 716.7 cM [29]. In June 2016, researchers performed de novo sequencing of pearl millet to identify genes associated with downy mildew and obtained 684.97 Mb of data and 1,295,196 high-quality reads [30]. In 2017, Varshney et al. [15] obtained a sketch of the pearl millet genome using whole genome shotgun and bacterial artificial chromosome sequencing. Concetta et al. (2018) discovered the origin of the pearl millet by sequencing the genome of 221 types of pearl millet, including wild-type and traditional varieties [31]. To the best of our knowledge, there have been no reports of the full-length transcriptome of the pearl millet in previous studies. In this study, we performed single-molecule long-reading sequencing of total RNA from 12 samples at four stages (three-leaf stage, five-leaf stage, flowering stage and heading stage) of pearl millet. In this study, we identified 132,488 transcripts (average length 3102 bp, N50 3302 bp) containing 63,090 more transcripts than the previously reported 69,998 (average length 725 bp, N501014 bp) transcripts in 2017 in same crop (Table 1). A total of 64,878 genes were identified, of which 26,299 were newly discovered. In this study, 27,190 (41.91%) genes were simultaneously annotated in seven databases (Nt, Nr, Pfam, KOG, Swiss-prot, KEGG, and GO), and 62,436 (96.24%) genes were annotated in at least one database. We annotated 59,981 genes by KEGG, which is 38,526 more genes (21,455 genes) than had been previously reported. We annotated 51,764 genes with SwissProt, 30,238 more genes than the 21,455 that had been previously identified. This information provides more resources for the identification and uncovering of important genes in pearl millet. We found that 1507 DEGs were up-regulated or down-regulated under heat stress and drought stress. We suggest that researchers study these genes in more detail, which are not only related to heat resistance but also to drought resistance.

### Expression of classical heat-related genes in pearl millet

Previously published literature revealed that pearl millet is highly resistant to heat stress, but no information regarding RNA sequencing exists. We performed RNA sequencing on heat-stressed pearl millet and discovered DEGs associated with reactive oxygen species (ROS) produced by exposure to heat stress. As a starting signal of plants, ROS prompts local tissues to respond to local abiotic stress stimulation, but the expression of the ROS genes are temporary [32, 33]. We identified nine DEGs associated with ROS production, two DEGs encoding amine oxidases (AOC), and the remaining seven DEGs encoding polyamine oxidases (PAO). With the exception of two genes that were up-regulated, the others were down-regulated (Additional file 4). These results indicated that a variety of genes involved in ROS production were inhibited after 48 h of heat stress. This is consistent with previous studies of the expression of genes involved in ROS production that have not been continuously expressed under stress [14]. ROS scavenging enzymes play an important role in protecting plants from temperature stress and are essential for the detoxification of ROS produced under stress conditions [34]. We found that 10 DEGs encoded ROS scavenging enzymes, five of which were up-regulated (Additional file 4), while the other five genes were down-regulated. These data suggest that when ROS is reduced, ROS clearance enzymes will decrease, and this effect is not sustained. The accumulation of heat shock proteins (HSP) under the control of heat stress transcription factors (HSFS) plays an important role in the heat stress response (HSR) and subsequently produces heat tolerance in plants and other organisms [35, 36]. In this study, we found that 30 DEGs were associated with HSPs; three DEGs encoded HSP100; 14 DEGs encoded HSP90; 12 DEGs encoded HSP70, and one DEG encoded sHSP, and all were up-regulated DEGs (Additional file 4). We hypothesize that the heat shock proteins still function even if the plants are subjected to sustained heat stress.

### Expression of typical drought-related genes in pearl millet

Under drought stress, the ABA content of plants increases, which regulates the opening and closing of pores, reduces water loss and maintains the moisture content of plants [37, 38]. Under drought stress, we found five DEGs encoding 9-cis-epoxycarotenoid dioxygenases (NCED), 1 DEG encoding zeaxanthin epoxidase (ABA1), and two DEGs encoding short-chain dehydrogenases/reductases (ABA2), four of which were up-regulated DEGs, and the other four were down-regulated DEGs (Additional file 5). We also discovered a DEG encoding PYL8, which is a receptor for ABA. The gene was down-regulated under drought stress (Additional file 5). This indicates that under 48 h of drought stress, the ABA pathway still functions in the drought stress response. Previous studies have found that slow anion channel correlation (SLAC) regulates the opening and closing of stomata, which is necessary for stomatal closure [39]. After 48 h of drought stress, a SLAC-encoding DEG was identified, which indicates that even after prolonged drought stress, pearl millet reduces water evaporation by regulating stomatal closure. (Additional file 5). The *Asr* gene family is classified as a new group of late embryogenic abundant (LEA) genes and is involved in adaptation to drought stress [40]. Under stress condition, the expression of the *Asr* gene was up-regulated. We found two *Asr* genes with different levels of expression, which were up-regulated under drought stress and are consistent with previous reports. (Additional file 5).

### In the same molecular mechanism, the pearl millet selects different proteins to respond to different stresses

The results showed that 5039 DEGs and 4603 DEGs were regulated under heat stress and drought stress, respectively, indicating that plants have different molecular mechanisms for heat and drought stress [41]. In addition, even if the same mechanism exists under both types of stresses, the types of proteins involved, the number of proteins, and the choice of related genes are different, such as ROS clearance mechanisms. Under drought stress, we found 14 DEGs associated with ROS scavenging enzymes, two DEGs encoding SOD, 11 DEGs encoding APX, and one DEG encoding GPX, six of which were up-regulated DEGs, while the other eight DEGs were down-regulated. The gene encoding CAT was significantly expressed 48 h after heat stress, but the expression of the gene was not significant under drought stress. The number of DEGs encoding APX proteins was higher under drought stress than heat stress. The number and type of ROS scavenging enzymes expressed under drought stress were different than those expressed from heat stress, indicating that ROS scavenging enzymes are specific to the type of stress (Table 2). A total of 1881 DEGs were collectively expressed under heat stress and drought stress, indicating that these 1881 DEGs play important roles under both types of stresses. Among the 1881 DEGs, 1577 DEGs with the same pattern of expression (both up-regulated or down-regulated under heat stress and drought stress) are essential to combat heat stress and drought stress, such as HSPs. HSPs are essential molecular chaperones in eukaryotic cells that play an important role in the folding and activation of proteins involved in signal transduction and regulation of the cell cycle. Under drought stress, we found that 26 DEGs encode HSP, of which 11 DEGs encode HSP70, 14 encode HSP90, and one encodes sHSP, all of which up-regulate DEGs (Table 3). Among them, 12 DEGs encoding HSP existed in both heat stress and drought stress. There were 304 DEGs with different expression patterns under heat stress and drought stress, indicating that different stresses have different levels of regulation of the same gene.

**Table 2:** Expression of gene encoding peroxide scavenging enzyme under heat stress and drought stress

annotation	Heat stress		drought stress	
	Gene	log2(HS/CK)	Gene	log2(DS/CK)
SOD	*i0_LQ_LWC_c2218/f1p0/833*	1.1734		
			*i2_HQ_LWC_c7659/f3p1/2700*	1.8944
			*i1_LQ_LWC_c42429/f1p0/1061*	1.5801
GPX	*i0_HQ_LWC_c63/f5p0/847*	−1.3626		
			*i1_LQ_LWC_c42213/f1p2/1035*	−1.4874
CAT	*i1_LQ_LWC_c20347/f1p0/1948*	−4.4651		
	*i2_HQ_LWC_c41068/f2p7/2070*	1.6112		
	*i1_LQ_LWC_c12593/f1p0/1872*	−3.7907		
	*i2_HQ_LWC_c4824/f3p1/2226*	−4.5763		
APX	*i1_LQ_LWC_c18498/f1p3/1627*	2.9239		
	*i1_LQ_LWC_c38392/f1p0/1178*	−1.3366		
	*i3_LQ_LWC_c37944/f1p0/3280*	1.2566		
	*i1_LQ_LWC_c22741/f1p1/1544*	1.7925	*i1_LQ_LWC_c22741/f1p1/1544*	−2.7765
			*i1_HQ_LWC_c28271/f15p0/1733*	−1.3777
			*i1_HQ_LWC_c40231/f3p0/1461*	3.1134
			*i1_LQ_LWC_c13218/f1p0/1219*	1.4302
			*i1_LQ_LWC_c13821/f1p5/1427*	−1.2859
			*i1_LQ_LWC_c18173/f1p0/1818*	−2.3155
			*i1_LQ_LWC_c18294/f1p0/1406*	−1.5224
			*i1_LQ_LWC_c26053/f1p6/1719*	−2.2266
			*i1_LQ_LWC_c36589/f1p0/1714*	−1.9296
			*i1_LQ_LWC_c38632/f1p0/1092*	1.4572
			*i2_LQ_LWC_c65698/f1p0/2629*	2.4454

Note: *HS* heat treatment, *CK* control, *DS* drought treatment

**Table 3:** Expression of gene encoding heat shock protein under heat stress and drought stress

Annotation	Heat stress		drought stress	
	Gene	log2(HS/CK)	Gene	log2(DS/CK)
sHSP	*i0_LQ_LWC_c967/f1p0/765*	4.9898	*i0_LQ_LWC_c967/f1p0/765*	10.152
HSP 70			*i1_LQ_LWC_c32521/f1p1/1976*	
			*i1_LQ_LWC_c22890/f1p0/1926*	
			*i2_LQ_LWC_c53787/f1p5/2383*	5.0347
			*i2_LQ_LWC_c86046/f1p8/3023*	1.3889
			*i3_HQ_LWC_c31973/f15p0/3104*	1.0617
	*i2_HQ_LWC_c43630/f6p12/2432*	5.3173	*i2_HQ_LWC_c43630/f6p12/2432*	6.901
	*i2_LQ_LWC_c104760/f1p1/2446*	1.1635	*i2_LQ_LWC_c104760/f1p1/2446*	1.7534
	*i2_LQ_LWC_c3333/f1p2/2529*	4.6887	*i2_LQ_LWC_c3333/f1p2/2529*	5.7184
	*i2_LQ_LWC_c81928/f1p8/2468*	3.0743	*i2_LQ_LWC_c84968/f1p1/2090*	1.5487
	*i2_LQ_LWC_c84968/f1p1/2090*	1.2513	*i3_LQ_LWC_c21708/f1p2/3195*	1.3274
	*i3_LQ_LWC_c21708/f1p2/3195*	1.2128	*i3_LQ_LWC_c27490/f1p0/3150*	6.7401
	*i3_LQ_LWC_c27490/f1p0/3150*	4.1929		
	*i2_HQ_LWC_c127192/f35p1/2517*	1.2971		
	*i2_HQ_LWC_c55656/f2p12/2212*	1.9817		
	*i2_HQ_LWC_c59895/f32p11/2345*	1.6303		
	*i2_HQ_LWC_c69799/f2p1/2652*	2.5412		
	*i2_LQ_LWC_c22856/f1p14/2305*	2.0376		
HSP 90			*i2_HQ_LWC_c127285/f2p25/2794*	1.4443
			*i2_HQ_LWC_c34808/f2p10/2596*	1.4052
			*i2_HQ_LWC_c97463/f19p4/2142*	1.4033
			*i2_LQ_LWC_c104439/f1p16/2591*	1.9891
			*i2_LQ_LWC_c3878/f1p2/2975*	1.7125
			*i2_LQ_LWC_c64587/f1p17/2400*	2.3499
			*i2_LQ_LWC_c8803/f1p15/2771*	2.3145
			*i1_LQ_LWC_c13864/f1p0/1351*	1.279
			*i2_LQ_LWC_c25038/f1p7/2643*	2.0169
	*i2_HQ_LWC_c49563/f2p1/2825*	3.0512	*i2_HQ_LWC_c49563/f2p1/2825*	3.2962
	*i2_HQ_LWC_c50337/f2p2/2836*	1.0389	*i2_HQ_LWC_c50337/f2p2/2836*	1.6639
	*i2_LQ_LWC_c106886/f1p1/2786*	2.3794	*i2_LQ_LWC_c106886/f1p1/2786*	2.7368
	*i2_LQ_LWC_c90259/f1p14/2485*	2.9896	*i2_LQ_LWC_c90259/f1p14/2485*	2.9776
	*i4_LQ_LWC_c19908/f1p0/5045*	3.0399	*i4_LQ_LWC_c19908/f1p0/5045*	3.1358
	*i2_LQ_LWC_c121080/f1p8/2511*	7.2738		
	*i3_LQ_LWC_c19538/f1p2/3645*	2.4181		
	*i2_LQ_LWC_c11597/f1p0/2377*	2.1599		
	*i2_LQ_LWC_c126787/f141p12/2519*	1.627		
	*i2_LQ_LWC_c33264/f1p2/2930*	2.7331		
	*i2_LQ_LWC_c33469/f1p1/2516*	3.2167		
HSP100	*i2_LQ_LWC_c102911/f1p4/3026*	2.213		
	*i2_LQ_LWC_c12834/f1p3/2671*	1.5904		
	*i2_LQ_LWC_c88848/f1p1/2115*	3.2613		

Note: *HS* heat treatment, *CK* control, *DS* drought treatment

## Conclusions

We combined two sequencing technologies to study the similarities and differences of the molecular mechanism of millet under heat and drought stress. In this study, 63,090 new transcripts and 26,299 new genes were identified, and the functional annotation of genes was improved by 20.18%. Under heat and drought stress, 6920 and 6484 genes were differentially expressed, respectively, and 1881 differentially expressed genes were present in both types of stresses. This information provides additional resources to identify and unearth important genes in pearl millet. In addition, we found that under the same mechanism of response, plants have different protein choices when faced with different types of stresses. This lays the foundation to study the heat and drought resistance mechanism of pearl millet.

## Methods

### Plant materials, cultivation and treatment

The pearl millet variety Tifleaf 3 used in this study was provided by Sichuan Agricultural University and is preserved in the Department of Grassland Science, College of Animal Science and Technology, Sichuan Agricultural University, Ya’an, Sichuan Province, China. Pearl millet seeds were grown in pots (10*15 cm) containing quartz sand and placed in a growth chamber. The pots were exposed to 14 h of light at 26 °C and 10 h of darkness at 22 °C for 13 days with 50% Hoagland’s nutrient solution (1 mM MgSO_4_, 1 mM KH_2_PO_4_, 1 mM NH_4_NO_3_, 0.5 mM CaCl_2_, 0.1 mM FeNa-EDTA, 25 mM NaCl, 0.1 mM H_3_BO_3_, 0.1 mM Na_2_SiO_3_, 1.5 μM CuSO_4_, 50 μM KCl, 10 μMMnSO_4_, 0.075 μMNa_2_MoO_4_ and 2 μM ZnSO_4_). Thirteen-day-old plants were divided into three groups (control, heat treatment group and drought treatment group). The culture conditions for the control group were 14 h of light at 26 °C and 10 h of darkness at 22 °C with 50% of Hoagland’s nutrient solution. The plants in the heat treatment group were exposed to 14 h of light at 40 °C and 10 h of darkness at 35 °C with 50% of Hoagland’s nutrient solution. The plants in the drought treatment group were exposed to 14 h of light at 26 °C and 10 h of darkness at 22 °C with 20% PEG (polyethylene glycol 6000) dissolved in 50% of Hoagland’s nutrient solution. All the treatment time is 48 h (2 days).

### RNA preparation for Iso-Seq

Leaf and root samples of the treated plants were collected separately at the three-leaf and five-leaf stages. When the pearl millet was in the heading stage, the spikelet, leaves, stems and roots were collected. While at the flowering stage, the spikelet, leaves, stems and roots were collected, and these samples were immediately stored at − 80 °C. The RNA was extracted using the RNeasy Plant Mini Kit [42], and the RNA quality was analysed using RNA gel electrophoresis. Equal amounts of RNA from 12 samples (1 μg per sample) were pooled together to form total RNA, and then the SMRT library was prepared using 3 μg total RNA.

### PacBio Iso-Seq library preparation and sequencing

The Iso-Seq library was prepared using the Isoform Sequencing protocol (Iso-Seq) with the Clontech SMARTer PCR cDNA Synthesis Kit and the BluePippin Size Selection System protocol as described by Pacific Biosciences (P/N100–377–100-05 and P/N100–377–100-04) with some modifications. First, 3 μl of total RNA solution was added to deionized water containing a single primer and incubated at 72 °C for 3 min (3′ SMART primer IIA from the Clontech SMARTer kit 5′–AAGCAGTGGTATCAACGCAGAGTACTNN–3′). Next, the SMARTER II A oligonucleotide (from the Clontech SMARTer kit 5′-AAGCAGTGGTATCAACGCAGAGTACXXXXX–3′), 5X first-strand buffer, DTT, dNTP mix, RNase inhibitor and SMARTScribe reverse transcriptase were added to the mixture and incubated at 72 °C for 1 h. Finally, the reaction was terminated at 70 °C. After 23 PCR cycles, the length of the PCR product was screened by the BluePippin Size Selection System and divided into 1–2 Kb, 2–3 Kb and 3–10 Kb fragments. After size screening, the cDNA was subjected to 12 cycles of PCR reactions. The PCR amplification products were used to construct the SMRTbell Template libraries using the Iso-Seq protocol. The libraries were prepared for sequencing by annealing a sequencing primer (component of the SMRTbell Template Prep Kit 1.0) and binding polymerase to the primer-annealed template.

### Iso-Seq data analysis

Processing of raw data was conducted by SMRTlink 5.1 software (https://www.pacb.com/videos/tutorial-minor-variant-analysis-smrt-link-v5-0-0/. The parameter settings were min_length 50, max_drop_fraction 0.8, no_polish TRUE, min_zscore − 9999.0, min_passes 2, min_predicted_accuracy 0.8, max_length 15,000, generating a cyclic consensus sequence from the sub read BAM file. Pbclassify.py, ignorepolyA false, minSeqLength 200 were used to divide the samples into full length and non-full length reads. Non-full length and full-length FASTA files were produced and fed into the cluster step, which performs isoform-level clustering (ICE), followed by final Arrow polishing, hq_quiver_min_accuracy 0.99, bin_by_primer false, bin_size_kb 1, qv_trim_5p 100, qv_trim_3p 30. Since the frequency of nucleotide indels and mismatches in the Iso-Seq reads were much higher than those in the shorter high-throughput sequencing, LoRDEC software was used to correct additional nucleotide errors in consensus reads based on Illumina RNA-Seq data. The redundancy in the corrected readings was obtained using software CD-HIT (−c 0.95 -T 6 -G 0 -aL 0.00 -aS 0.99) to obtain the final transcript for subsequent analysis.

### Gene functional annotation

We used the following five databases to align all predicted protein coding sequences, NCBI non-redundant protein (NR, cutoff E value ≤1^e-5^), NCBI non-redundant nucleotide (NT, E value ≤1^e-5^), Swiss-Prot protein (http://www.expasy.org/sprot/, cutoff E value ≤1^e-5^), KOG (http://www.ncbi.nlm.nih.gov/COG/, cut-off E value ≤1^e-3^) [43], protein family (Pfam: http://pfam.sanger.ac.uk/, cutoff E-value ≤0.01), KEGG (http://www.genome.jp/kegg, cut-off E value ≤1^e-5^) pathways [44]. Utilize Blast2GO (http://www.blast2go.com) program for GO annotation (http://www.geneontology.org) based on NR annotation (cutoff E-value ≤1^e-10^).

### CDS prediction

Protein coding sequences from cDNA were identified by the ANGEL pipeline (a long-read implementation of ANGLE). We used closely related species to ensure that the protein sequences were ANGEL-trained and then performed an ANGEL prediction for a given sequence.

### TF analysis and Lnc-RNA analysis

Transcription factors were predicted by iTAK software.

We predicted lncRNA using four software: a) The CNCI (Coding-Non-Coding-Index, parameters as default parameters) was an effective software to distinguish the protein-encoding and non-coding sequences by profiles adjoining nucleotide triplets without relying on known annotations. b) The CPC (Coding Potential Calculator) was used to assess the ORF extent and quality of transcripts and search the sequenced base eukaryote protein database from NCBI (e value “1^e-10^”) to identify coding transcripts and non-coding transcripts. c) All the transcripts were translated, and then Pfam Scan (−E 0.001 --domE 0.001) was used to determine if they have a domain of a known protein family. d) Predicting Lnc-RNA with PLEK SVM classifier (−minlength 200). The PLEK SVM classifier identifies transcripts of encoded proteins by optimizing the K-mer approach, which eliminates the need for reference genomes and annotations. All of the four software programs described above identified non-coding transcripts, which were identified as Lnc-RNA.

### RNA preparation for RNA-Seq

Three treatments were conducted simultaneously. After 48 h of treatment, we randomly selected the leaves of 16 seedlings and collected them in a cryogenic vials and immediately stored at − 80 °C (Additional file 1). Three biological replicates were set for each treatment. The RNA was extracted from the samples using the RNeasy Plant Mini Kit, and the RNA quality was checked using RNA gel electrophoresis.

### RNA-Seq library preparation and sequencing

The purity of RNA was detected using a NanoDrop spectrophotometer (California, USA), and the concentration of RNA was determined by a Qubit RNA assay kit in a Qubit 2.0 fluorometer system (California, USA). The library was constructed using the NEBNext® UltraTM Directional RNA Library Prep Kit for Illumina® (California, USA). The NEBNext®Poly (A) mRNA Magnetic Isolation Module was used to enrich the mRNA, and Fragmentation Buffer was added to break the mRNA into short segments. A strand of cDNA was synthesized with random hexamer primers. The second strand of cDNA was synthesized by adding buffer, dNTPs and DNA polymerase I. The double strand cDNA was purified by AMPure XP beads. The purified cDNA was repaired at the end; a tail was added and sequenced, and the fragment size was selected by AMPure XP beads. Finally, the final cDNA library was obtained by PCR enrichment. Qubit 2.0 was used for preliminary quantification; Agilent 2100 was used to detect the inserted fragments of the library, and finally Illumina Hi-Seq 2000 was used for sequencing. A total of 9 RNA-Seq libraries were established.

### Quantification of the gene expression levels

Identify the gene expression level of each sample by RSEM [45]. The clean data generated by Illumina sequencing were mapped to SMRT sequencing data, and the read count of each gene was obtained from the mapping results [46]. The read count value of each gene was converted to the FPKM value (Fragments per Kilobase Million), and genes with FPKM> 0.3 were selected for analysis.

### Identification and function analysis of DEGs

Differential expression analysis was performed using the DESeq R package (1.10.1) [47] to identify DEGs between the heat-stressed and control samples and between drought-stressed and control samples. DESeq is a statistical program that determines the differential expression in digital gene expression data using a model-based negative binomial distribution. The P rate was adjusted by the p.adjust function to control the error rate. The genes with an adjusted *P*-value < obtained by DESeq software were considered to be differentially expressed, and the significance of DEGs determined by the absolute value of log2 (Group1/Group2) ≥ 1 as the threshold.

We use the GOseq R package to perform GO enrichment analysis on DEGs. The software package is based on Wallenius non-central hypergeometric distribution, and estimates and adjusts the preference of DEGs length. Finally, the KEGG enrichment analysis of DEGs was carried out by KOBAS software [48].

## Supplementary information

**Additional file 1.** Pearl millet after 48 h of heat treatment, drought treatment and control treatment

**Additional file 2.** Photosynthesis-related gene expression under heat stress

**Additional file 3.** Expression of Glycerophospholipid metabolic related genes during drought stress

**Additional File 4.** DEGs expression under the heat stress of pearl millet

**Additional File 5.** DEGs expression under the drought stress of pearl millet

## Data Availability

The datasets supporting the conclusions of this article are included within the article (and its additional files). Sequencing database for pearl millet could download from NCBI under the accession number SRR11816223, and the data will be shared on reasonable request of the corresponding author.

## Abbreviations

DEG: Differentially expressed gene
GO: Gene Ontology
KEGG: Kyoto Encyclopedia of Genes and Genome
HSP: heat shock proteins
ROS: Reactive oxygen species
AOC: Amine oxidases
PAO: Polyamine oxidases
HSFS: Heat stress transcription factors
NCED: 9-cis-epoxycarotenoid dioxygenases
ABA1: Zeaxanthin epoxidase
ABA2: Short-chain dehydrogenases/reductases
SLAC: Slow anion channel correlation
SOD: Superoxide Dismutas
CAT: Catalase
apx: aseorbateperoxidase
LEA: Ate embryogenic abundant
