# Supplementary figures and images for: Transcriptome analysis of heat stress and drought stress in pearl millet based on Pacbio full-length transcriptome sequencing
Source: BMC Plant Biol. 2020 Jul 8;20:323. doi: 10.1186/s12870-020-02530-0 (PMC7346438; doi:10.1186/s12870-020-02530-0)

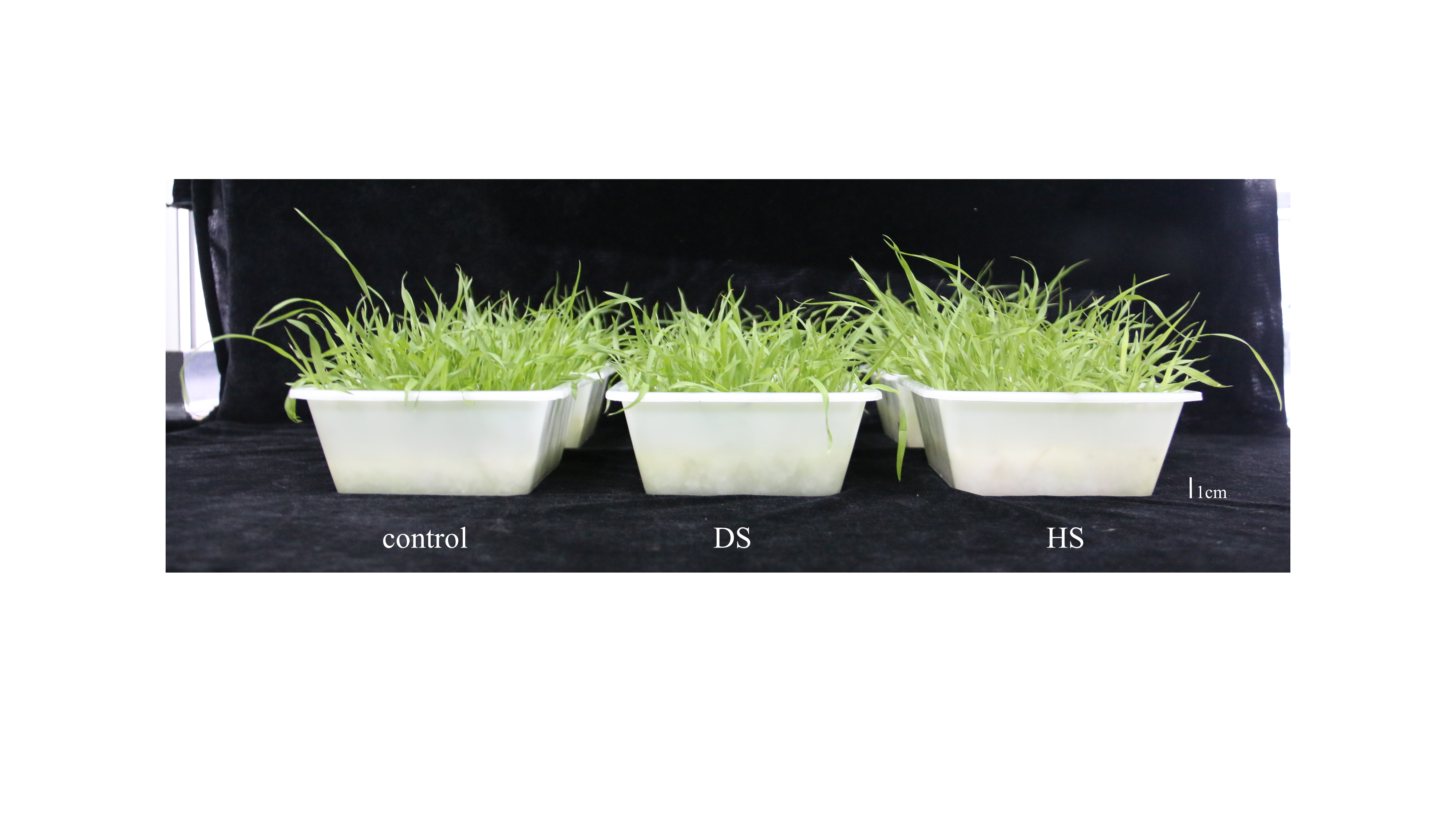

Supplement: Supplementary file 1 — Additional file 1. Pearl millet after 48 h of heat treatment, drought treatment and control treatment [file 12870_2020_2530_MOESM1_ESM.tif]
